# Local Positioning System Using Flickering Infrared LEDs

**DOI:** 10.3390/s17112518

**Published:** 2017-11-03

**Authors:** Thibaut Raharijaona, Rodolphe Mawonou, Thanh Vu Nguyen, Fabien Colonnier, Marc Boyron, Julien Diperi, Stéphane Viollet

**Affiliations:** 1 Aix Marseille University, CNRS, ISM, Marseille 13009 , France; Kodjo.Mawonou@supelec.fr (R.M.); ngthanh.vu94@gmail.com (T.V.N.); tslftac@nus.edu.sg (F.C.); marc.boyron@univ-amu.fr (M.B.); julien.diperi@univ-amu.fr (J.D.); stephane.viollet@univ-amu.fr (S.V.); 2 Temasek Labs, National University of Singapore, Singapore 117411, Singapore

**Keywords:** indoor positioning, infrared light communication, LED, photodiode (PD), visual motion

## Abstract

A minimalistic optical sensing device for the indoor localization is proposed to estimate the relative position between the sensor and active markers using amplitude modulated infrared light. The innovative insect-based sensor can measure azimuth and elevation angles with respect to two small and cheap active infrared light emitting diodes (LEDs) flickering at two different frequencies. In comparison to a previous lensless visual sensor that we proposed for proximal localization (less than 30 cm), we implemented: (i) a minimalistic sensor in terms of small size (10 cm${}^{3}$), light weight (6 g) and low power consumption (0.4 W); (ii) an Arduino-compatible demodulator for fast analog signal processing requiring low computational resources; and (iii) an indoor positioning system for a mobile robotic application. Our results confirmed that the proposed sensor was able to estimate the position at a distance of 2 m with an accuracy as small as 2-cm at a sampling frequency of 100 Hz. Our sensor can be also suitable to be implemented in a position feedback loop for indoor robotic applications in GPS-denied environment.

## 1. Introduction

In the absence of global positioning system (GPS), an indoor positioning system must be used to provide a local position of the autonomous robot in a constrained environment with dust, smoke and various lighting conditions (darkness, half-light, and flickering light).

There are numerous existing technical solutions for the indoor localization using: (i) infrastructure such as ultrasonic signals [1], ultra wideband technology [2] and fingerprinting approaches with wireless sensors networks [3]; and (ii) onboard sensors such as monocular cameras, stereo imaging and Light Detection and Ranging (LIDAR). For industrial applications inside warehouses in [4], automated guided vehicles (AGVs) localize themselves by triangulation based on reflector landmarks detected by laser scanners. In [5], visual odometry for localization aims at estimating the pose of a vehicle through examination of the changes that motion induces on the images acquired by onboard cameras. In [6], in conjunction with signals produced from inertial sensors and wheel encoders, a map of the magnetic field was used to precisely localize an indoor robot without any additional infrastructure. Moreover, the inertial sensors such as accelerometers, rate gyros combined magnetometers can be used to estimate the angular position, velocity or acceleration of a mobile robot. However, the effect of noise on the integrated signals strongly affects the position estimation and leads to difficulties in getting the information with a high precision over a large amount of time. In [7], in the context of visible light communication, an indoor positioning system using multiple optical receivers composed of photodiodes (PD) provides coordinates and orientation of the mobile receiver with an achievable position error less than 0.1 m. In [8], a linear positioning system based on infrared (IR) beacon aims at localizing indoors pedestrians using a trigonometrical survey. The IR beacon is attached to the shopping bag. The receiver installed in the ceiling at the height of 2.3 m is a PD array and measures the angle of incidence of the beacon ray. Therefore, the indoor position of the IR beacon is calculated and an identifier signal is sent to a computer for processing via wireless communication. In [9], using visible light communication, a novel indoor localization system is presented, where LED beacons determine the position of the target sensor, including a camera, an inclinometer, and a magnetometer. The localization is performed using geometric- and consensus-based techniques adapted to a high number of beacons and outliers. The tests presented show that the accuracy of the system is in the low decimeter range.

This paper proposes the development of a very different kind of indoor localization technique. A novel local positioning system is developed based on a bio-inspired optical and minimalistic sensor in terms of mass, size, cost and computational resources using photodiodes and flickering IR LEDs. As presented in [10], the comparison between charge couple device (CCD) image sensors, complementary metal oxide semiconductor (CMOS) image sensors and PDs pointed out the advantages of PDs in terms of speed, sensitivity, energy consumption and system complexity. Moreover, PD is easy to fabricate and has low production costs. As proposed in [11], LEDs offer advantageous properties such as reliability, lower power consumption, long lifetime and can be used as a communication device. For optical communications in free space under fog and smoke conditions, Ijaz et al. showed that near infrared light sources are the most robust wavelengths to link failure [12]. In [13], a lensless sensor was prototyped to estimate the position of active IR LEDs for proximal localization (up to 30 cm). To increase the operating range (≥1.5 m) the paper addresses a brand new design for indoor localization in 2D. Using PD and IR LEDs, our indoor positioning system embeds an innovative optical sensor robust to lighting conditions.

Section 2 introduces the computer-aided design of the sensor in 3D. The fabrication of a new tiny optical sensing device with a custom-made signal processing board shielded to an Arduino board is presented in Section 3. Section 4 gives a short description of the bio-inspired optical sensor modeling and the principle underlying the signal processing algorithm. In Section 5, indoor localization is performed for the estimation of position in 2D. The localization of a mobile robot in 2D was also tested with the new sensor device implemented in the feedback control loop for trajectory tracking purpose. Section 6 concludes the paper.

## 2. Sensor Design

**Figure 1:**
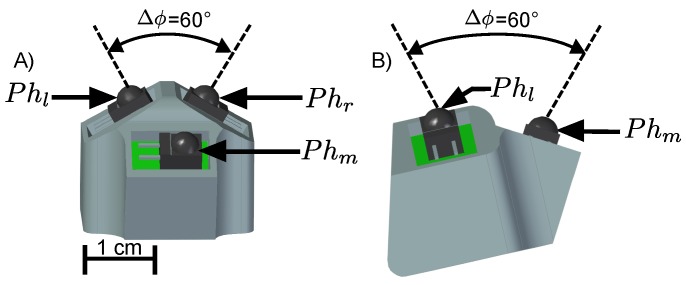
Computer-Aided Design of the optical sensor called HyperCube. Each side of the sensor consists of one photodiode soldered to a small printed circuit board ($Ph_{l}$, $Ph_{r}$, $Ph_{m}$ respectively): (**A**) top view; and (**B**) side view.

The optical sensor device, called HyperCube, designed and developed in this study, is equipped with three photodiodes (Figure 1). Each photodiode is mounted on the face of a tetrahedron. As depicted in Figure 1A,B, the optical axis of each photodiode is separated by an inter-receptor angle $\Delta \phi =60^{\circ }$, which defines the spatial acuity of the visual system [14].

## 3. Fabrication

**Figure 2:**
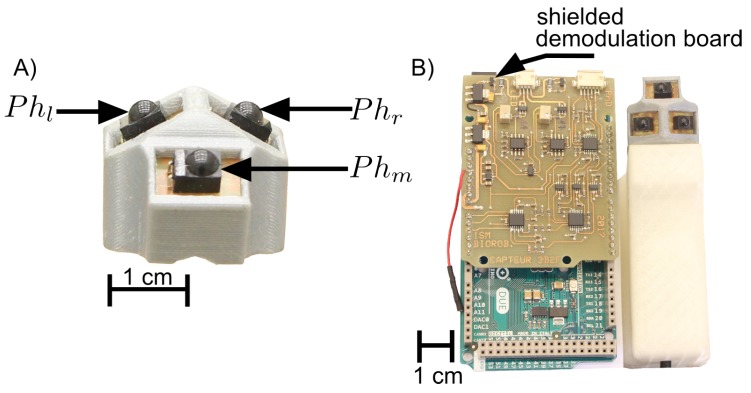
Description of the HyperCube hardware: (**A**) each photodiode is connected to an analog amplifier for the conversion of the photodiode current into an output voltage; and (**B**) the signal provided by the photodiodes is processed by a demodulation board shielded on an Arduino board.

The prototype of the sensor was obtained by 3D printing as shown on Figure 2A. It is equipped with three photodiodes made by Vishay Semiconductors with the reference **BPV22F**. Each photodiode has a maximum absorption at the wavelength of 950 nm which corresponds to the maximum emissive power of the infrared LEDs **SF4249**. As presented in Figure 2B, a custom-made electronic board for the frequency modulation of the flickering IR LEDs can produce two separate signals at 11 kHz and 17 kHz, respectively. The same electronic board composed of two analog demodulation circuits performs the acquisition and demodulation. Shielded on the Arduino board, the latter performs the visual signal processing and provides an estimation of the HyperCube’s angular position (i.e., azimuth and elevation angles) with respect to the infrared LEDs.

## 4. Modeling

Each photodiode features an angular sensitivity which is defined by the angle of acceptance denoted Δρ, i.e., the full width at half maximum of the angular sensitivity. A bell-shaped sensitivity function models the angular sensitivity of each photodiode. It was inspired by the Gaussian angular sensitivity function of flies’ photoreceptors as described in [15].

### 4.1. Angular Sensitivity of the Photosensors

**Figure 3:**
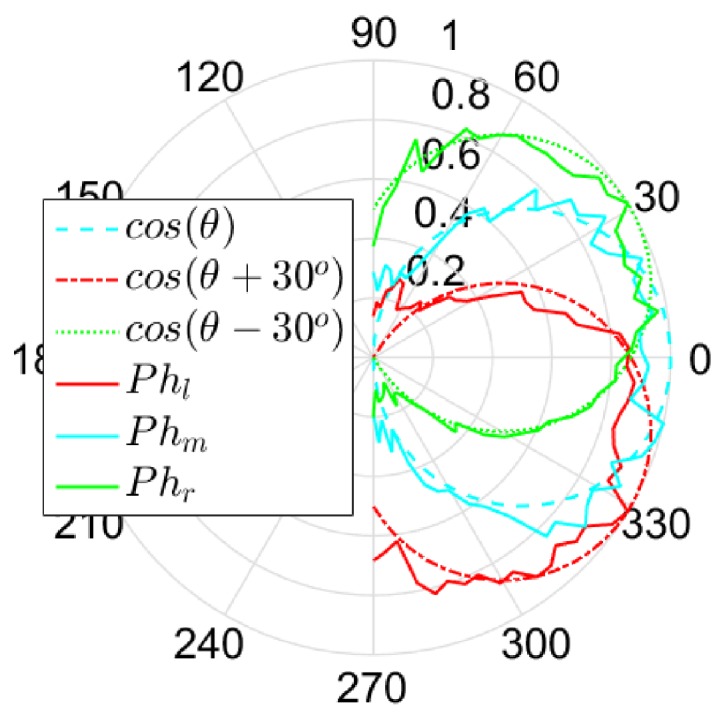
Sensor characterization and comparison between the theoretical (dotted lines) and measured angular sensitivities (continuous lines) of the photodiodes plotted here in polar coordinates.

As presented in Figure 3, the angular sensitivity of each photodiode in solid line is compared to the cosine-like angular sensitivity in dotted line. One can show that the cosine-like angular sensitivity of the model fits well to the experimental data and finally fits better than a Gaussian function. Moreover, the angle of acceptance Δρ is equal to $120^{\circ }$ and Δϕ between $Ph_{l}$ and $Ph_{r}$ is equal to $60^{\circ }$.

### 4.2. Principle of the Sensor

**Figure 4:**
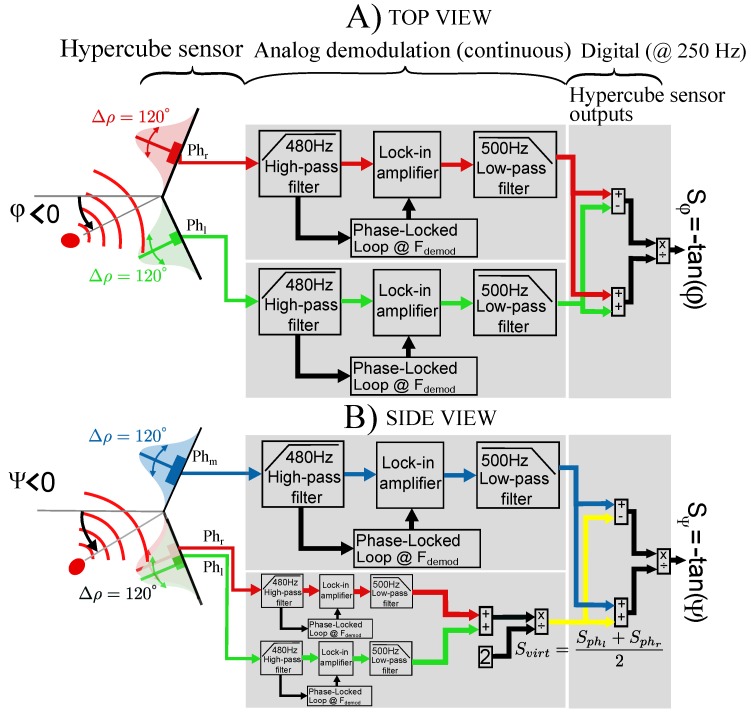
Sketch diagram of the signal processing algorithm. (**A**). Top view: The sensor measures the azimuth φ. The left part shows the IR LED modulated at a frequency noted $F_{demod}$ (11 kHz or 17 kHz). In this view, HyperCube is composed of two photosensors $Ph_{l}$ and $Ph_{r}$ with their respective cosine-like angular sensitivities (see Figure 3B). (**B**). Side View: The same signal processing is applied on the signal provided by the photosensor $Ph_{m}$ of HyperCube and a virtual photosensor where $S_{virt}=\frac{S_{ph_{l}}+S_{ph_{r}}}{2}$.

Two demodulated photodiode output signals are processed by an Arduino microcontroller. The analog demodulation steps were achieved by our custom-made shield board connected to the Arduino (see Figure 4). As shown in Figure 4A,B, the digital processing computes the relative difference over the sum of two adjacent demodulated photosensor output signals in order to assess the angular measurements (azimuth and elevation) [16]. The demodulation steps consist of using classical lock-in detection to demodulate the signal. However, the lock-in amplifier required a modulation signal which is provided here by a Phase-lock-loop circuit due to the fact that there is no physical link between the source (LED) and the receiver (photodiode).

**Figure 5:**
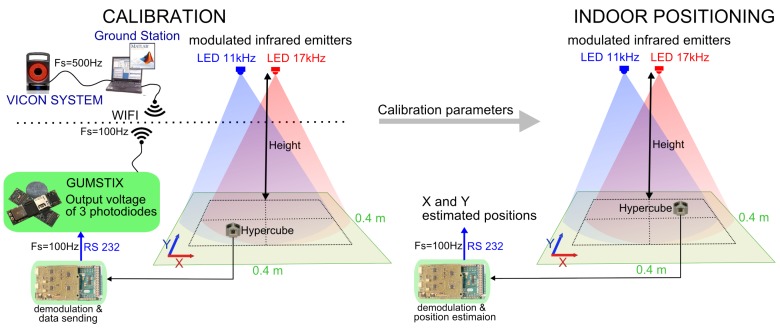
The principle of the indoor positioning solution at a fixed height consists of two steps: (i) the calibration procedure aims at finding the parameters which minimize the quadratic error metrics between the reference position given by the Vicon system and the data provided by the sensor; and (ii) given the calibration parameters, the sole use of the sensor device connected to the shielded demodulation board allows to estimate the positions X and Y at a fixed height.

As depicted in Figure 4, the digital processing operated in the microcontroller returns an output signal $S_{\varphi }$ for the azimuth φ with $S_{\varphi }=\frac{S_{ph_{r}}-S_{ph_{l}}}{S_{ph_{r}}+S_{ph_{l}}}$ and an output signal $S_{\psi }$ for the elevation ψ with $S_{\psi }=\frac{S_{ph_{m}}-S_{ph_{virt}}}{S_{ph_{m}}+S_{ph_{virt}}}$ where $S_{virt}=\frac{S_{ph_{l}}+S_{ph_{r}}}{2}$. According to the visual sensor model mentioned in [13], $S_{\varphi }\propto -tan\left(\varphi \right)$ and $S_{\psi }\propto -tan\left(\psi \right)$. Therefore, the relative position $\left(\hat{X},\hat{Y}\right)$ of the sensor with respect to the IR LED can be estimated with $\hat{X}=-tan\left(\varphi \right)\hat{Z}$ and $\hat{Y}\propto -tan\left(\psi \right)\hat{Z}$, where $\hat{Z}$ is the *a priori* known fixed height as shown on Figure 5.

## 5. Experimental Results

### 5.1. Position Estimation in 2D

A calibration, which consists of adjusting the sensor outputs $S_{\varphi }$ and $S_{\psi }$ to the ratios X/Z and Y/Z, is processed using the Vicon system.

(1) $$\begin{array}{cc} \mathrm{minimize} & \frac{1}{n}\sum \sqrt{{\left(X_{ref}-\hat{X}\right)}^{2}+{\left(Y_{ref}-\hat{Y}\right)}^{2}}\\ \mathrm{subject}\;\mathrm{to} & -\frac{X_{ref}}{\hat{Z}}=a_{\varphi 1}.S_{\varphi }^{2}+a_{\varphi 2}.S_{\varphi }+a_{\varphi 3}.S_{\psi }^{2}+a_{\varphi 4}.S_{\psi }+b_{\varphi }\\ & -\frac{Y_{ref}}{\hat{Z}}=a_{\psi 1}.S_{\varphi }^{2}+a_{\psi 2}.S_{\varphi }+a_{\psi 3}.S_{\psi }^{2}+a_{\psi 4}.S_{\psi }+b_{\psi } \end{array}$$

The coefficients $a_{\varphi i}$ and $a_{\psi i}$ are determined using Matlab^®^. The optimization Criterion (1) is to minimize the mean square error between the reference values and the actual data values provided by the sensor. The Matlab^®^ function *fminunc* is used to compute the coefficients. The localization was tested indoors for several lighting conditions. The optical sensing device was moving in *X* and *Y* at a fixed height as presented in Figure 5. After the calibration, the indoor localization is performed with the sole use of the optical sensor device connected to the demodulation board and the Arduino board.

**Figure 6:**
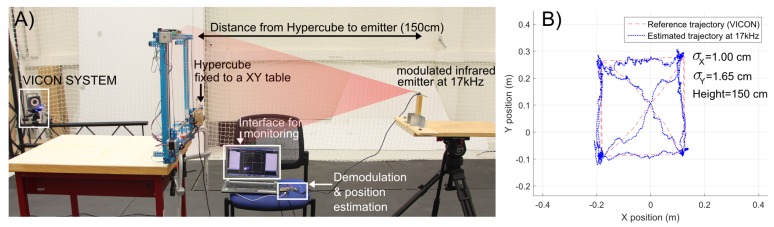
2D localization of HyperCube moved by hand using a *XY* table with respect to a fixed IR LED flickering at 17 kHz placed ahead of the sensor at a distance of 150 cm. The position estimation is based on the parameters obtained in the calibration procedure: (**A**) experimental setup; and (**B**) experimental results. The standard deviation for *X* estimation is only 1 cm and the standard deviation for *Y* estimation is 1.65 cm.

Figure 6A gives a description of the experimental setup. One can see the location of the modulated infrared emitter and the coverage of the system. In this experiment, HyperCube is fixed to a *XY* table and moved by hand. One can note from the indoor localization results (Figure 6B) that the position estimation at the fixed height of 150 cm is accurate. The precision obtained features a standard deviation about 1 cm for *X* and inferior to 2 cm for *Y* measurements. Experiments over a longer distance are presented in the next section.

### 5.2. Localization of a Mobile Robot in 2D

**Figure 7:**
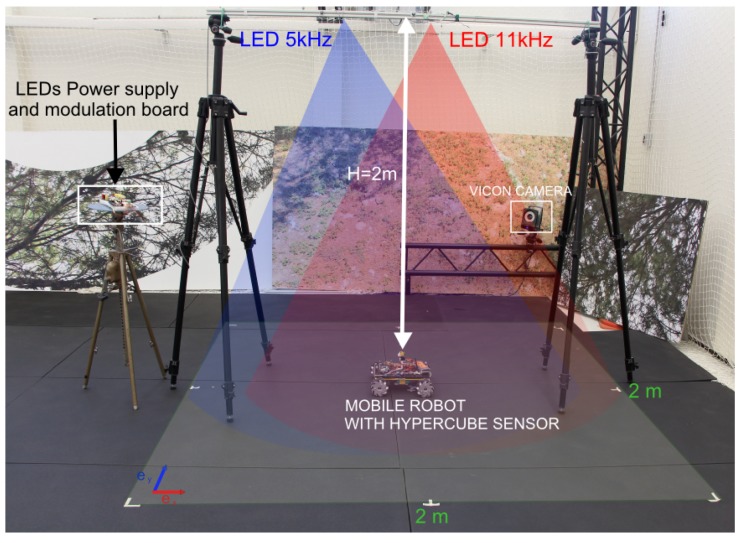
Picture of the experimental setup inside the motion capture system. The aim is to localize in 2D the mobile robot using HyperCube. The ground thruth is given by the Vicon cameras. The mecanum wheeled omni-directional robot is equipped with HyperCube, the localization coverage area is 2 m × 2 m. Two IR LEDs flickering at 5 kHz and 11 kHz were fixed on a horizontal bar placed above the robot at a height of 2 m.

In this section, we present the results obtained using HyperCube for indoor localizatin in 2D of a mobile robot. A new custom-made electronic board for the frequency modulation of the flickering IR LEDs is built. It can produce two separate signals at 5 kHz and 11 kHz. The mobile robot is equipped with a new custom-made electronic board composed of two analog demodulation circuits in charge of acquisition and demodulation. The experimental setup is presented in Figure 7. The ground height is H=2 m and the localization coverage area is 2 m × 2 m.

#### 5.2.1. Kinematics and Dynamics Modeling of the Mobile Robot

**Figure 8:**
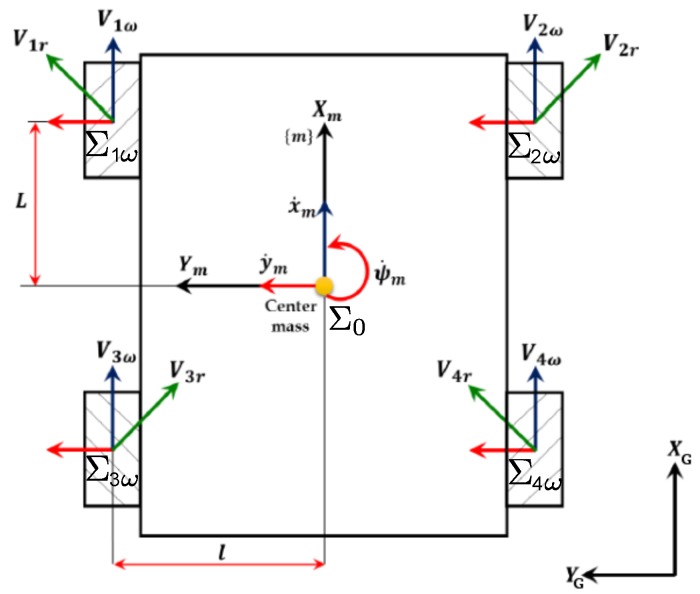
Disposition of the mecanum wheels and the frames.

Complete kinematics and dynamics modeling of the omni-directional robot with mecanum wheels are detailed in [17,18]. Figure 8 shows the disposition of the wheels related to the frames $\Sigma_{0}$, $\Sigma_{i\omega }$, (*i* = 1, 2, 3, 4). We define: $V_{i\omega }$ (*i* = 1, 2, 3, 4) the velocity vector corresponding to the wheels revolutions where $V_{i\omega }=R_{w}\times \omega_{i}$. $R_{w}$ is the radius of the wheel and $\omega_{i}$ is the revolution velocity of the wheel. $V_{ir}$ (*i* = 1, 2, 3, 4) is the tangential velocity vector of the free roller touching the floor and $V_{0}={\left[{\dot{x}}_{m}\;{\dot{y}}_{m}\;{\dot{\psi }}_{m}\right]}^{T}$ is the velocity vector in the local frame $\left(X_{m},Y_{m},Z_{m}\right)$.

The state vector $X={\left[x\;y\;\psi \right]}^{T}$ is composed of the positions *x*, *y* and the heading ψ in the global frame $\left(X_{G},Y_{G},Z_{G}\right)$. Kinematics equation describing the relationship between $V_{\omega }$ and $V_{0}$ is given by:

(2) $$V_{\omega }=\;J_{0}\cdot \;V_{0}$$

$J_{0}=\left[\begin{array}{ccc} 1 & -1 & -(l+L)\\ 1 & 1 & \left(l+L\right)\\ 1 & 1 & -(l+L)\\ 1 & -1 & \left(l+L\right) \end{array}\right]\in R^{4\times 3}$ is a transformation matrix and $V_{\omega }={\left[V_{1\omega }\;V_{2\omega }\;V_{3\omega }\;V_{4\omega }\right]}^{T}$ is the wheel velocity vector corresponding to the angular velocity. Oppositely, the mobile robot velocity can be derived from the wheel velocity using a pseudo inverse matrix as in (3):

(3) $$V_{0}={J_{0}}^{+}\cdot V_{\omega }$$

where ${J_{0}}^{+}={\left({J_{0}}^{T}\cdot \;J_{0}\right)}^{-1}{J_{0}}^{T}$. As a result, each element of $V_{0}$ is given by the following equations:

(4) $${\dot{x}}_{m}=\frac{R_{w}}{4}\left(\omega_{1}+\omega_{2}+\omega_{3}+\omega_{4}\right)$$

(5) $${\dot{y}}_{m}=\frac{R_{w}}{4}\left(-\omega_{1}+\omega_{2}+\omega_{3}-\omega_{4}\right)$$

(6) $${\dot{\psi }}_{m}=\frac{R_{w}}{4(L+l)}\left(-\omega_{1}+\omega_{2}-\omega_{3}+\omega_{4}\right)$$

The velocity $\dot{X}$ in the global frame $\left(X_{G},Y_{G},Z_{G}\right)$ is expressed in (7):

(7) $$\left[\begin{array}{c} \dot{x}\\ \dot{y}\\ \dot{\psi } \end{array}\right]=\left[\begin{array}{ccc} \cos(\psi ) & -\sin(\psi ) & 0\\ \sin(\psi ) & \cos(\psi ) & 0\\ 0 & 0 & 1 \end{array}\right]\cdot \left[\begin{array}{c} {\dot{x}}_{m}\\ {\dot{y}}_{m}\\ {\dot{\psi }}_{m} \end{array}\right]$$

As presented in [18,19], the vehicle dynamics is given by (8):

(8) $$\left[\begin{array}{c} \dot{\theta }\\ \ddot{\theta } \end{array}\right]=\left[\begin{array}{cc} 0_{4\times 4} & I_{4\times 4}\\ 0_{4\times 4} & -M^{-1}\;D_{\theta } \end{array}\right]\left[\begin{array}{c} \theta \\ \dot{\theta } \end{array}\right]+\left[\begin{array}{c} 0_{4\times 4}\\ M^{-1} \end{array}\right]\tau$$

$\theta ={\left[\theta_{1}\;\theta_{2}\;\theta_{3}\;\theta_{4}\right]}^{T}$ is the vector of the angular position of each wheel. $\tau ={\left[\tau_{1}\;\tau_{2}\;\tau_{3}\;\tau_{4}\right]}^{T}$ is the control input vector composed of the torque applied to each wheel with: $M=\left[\begin{array}{cccc} A+B+I_{w} & -B & B & A-B\\ -B & A+B+I_{w} & A-B & B\\ B & A-B & A+B+I_{w} & -B\\ A-B & B & -B & A+B+I_{w} \end{array}\right]$, $A=\frac{m{R_{w}}^{2}}{8}$, $B=\frac{I_{z}{R_{w}}^{2}}{16{\left(L+l\right)}^{2}}$ and $D_{\theta }$ is the coefficient of the wheel’s viscous friction. $I_{z}$ is the vehicle moment of inertia around the *Z* axis. $I_{w}$ is the wheel’s moment of inertia around the center of revolution.

#### 5.2.2. Design of Position Control

The standard robot motion control have been designed using a sliding mode dynamic controller to track a desired trajectory as detailed in [19]. Let us consider the state vector $X={\left[x_{11},\;x_{12},\;x_{21},\;x_{22},\;x_{31},\;x_{32}\right]}^{T}={\left[\int x,\;x,\;\int y,\;y,\;\int \psi ,\;\psi \right]}^{T}$ and the control input vector $u={\left[u_{1}\;u_{2}\;u_{3}\right]}^{T}={\left[{\dot{x}}_{m}\;{\dot{y}}_{m}\;{\dot{\psi }}_{m}\right]}^{T}$. From (7), the state space representation of the system is given by:

(9) $$\begin{array}{cc} {\dot{x}}_{11} & =x_{12}\\ {\dot{x}}_{12} & =u_{1}\cos\left(x_{32}\right)-u_{2}\sin\left(x_{32}\right)\\ {\dot{x}}_{21} & =x_{22}\\ {\dot{x}}_{12} & =u_{1}\sin\left(x_{32}\right)+u_{2}\cos\left(x_{32}\right)\\ {\dot{x}}_{31} & =x_{32}\\ {\dot{x}}_{32} & =u_{3} \end{array}$$

The control input vector $r={\left[r_{1}\;r_{2}\;r_{3}\right]}^{T}$ is defined to compensate for the nonlinear terms in (9)

(10) $$\begin{array}{cc} u_{1} & =\cos\left(x_{32}\right)r_{1}+\sin\left(x_{32}\right)r_{2}\\ u_{2} & =-\sin\left(x_{32}\right)r_{1}+\cos\left(x_{32}\right)r_{2}\\ u_{3} & =r_{3} \end{array}$$

Using (10) in the state space representation (9), a system of equations is written as following:

(11a) $$\begin{array}{c} \sum_{1}=\left\{\begin{array}{cc} {\dot{x}}_{11} & =x_{12}\\ {\dot{x}}_{12} & =r_{1} \end{array}\right. \end{array}$$

(11b) $$\begin{array}{c} \sum_{2}=\left\{\begin{array}{cc} {\dot{x}}_{21} & =x_{22}\\ {\dot{x}}_{12} & =r_{2} \end{array}\right. \end{array}$$

(11c) $$\begin{array}{c} \sum_{3}=\left\{\begin{array}{cc} {\dot{x}}_{31} & =x_{32}\\ {\dot{x}}_{32} & =r_{3} \end{array}\right. \end{array}$$

The Equation (11a,b) stand for the equations of translation and the Equation (11c) describes the movement of rotation. As defined in [19], the positions of reference $\xi_{1d}$ and $\xi_{2d}$ in the inertial frame are introduced. The orientation reference in the same frame is also noted $\xi_{3d}$. Therefore, the following subsystem of equations is written:

(12a) $$\begin{array}{c} \sum_{i}^{e}=\left\{\begin{array}{cc} {\dot{z}}_{i1} & =z_{i2}\\ {\dot{z}}_{i2} & =r_{i}-{\dot{\xi }}_{id}\;\forall i\in \left\{1,2,3\right\}\\ {\dot{\phi }}_{id} & =\xi_{id} \end{array}\right. \end{array}$$

with $z_{i1}=x_{i1}-\phi_{id}$, $z_{i2}=x_{i2}-\xi_{id}\;\forall i\in \left\{1,2,3\right\}$. The saturation function denoted $\sigma_{M}:R\to \;R$ is defined as:

(13a) $$\begin{array}{c} \sigma_{M}\left(S\right)=\left\{\begin{array}{c} S\;if\;|S|\;<\;M\\ sign(S)\times \;M\;otherwise \end{array}\right. \end{array}$$

The sliding surfaces $S_{i1}$ and $S_{i2}$ are defined for each axis *X* and *Y* such that:

(14) $$\begin{array}{cc} S_{i1} & =a_{i1}a_{i2}z_{i1}-a_{i2}z_{i2}\\ S_{i2} & =a_{i1}z_{i2} \end{array}$$

where the coefficients $a_{i1}$ and $a_{i2}$ are chosen to ensure the attractiveness of the sliding surfaces. Therefore, we propose the candidate Lyapunov functions $V_{i1}$ and $V_{i2}$ for each sliding surface:

(15) $$\begin{array}{cc} V_{i1} & =S_{i1}^{2}\\ V_{i2} & =S_{i2}^{2} \end{array}$$

As explained in [19], the exponential stability of the system is ensured and the control input signal $r_{i}$ can be written as:

(16) $$\begin{array}{c} r_{i}=\sigma_{i3}\left({\dot{\xi }}_{id}-\sigma_{M_{i2}}\left(a_{i1}z_{i2}+\sigma_{M_{i1}}\left(a_{i2}z_{i2}+a_{i1}a_{i2}z_{i1}\right)\right)\right)\;\forall i\in \left\{1,2,3\right\}. \end{array}$$

Using (10) and the expression provided by (16), the control input signals $u_{1}$, $u_{2}$ and $u_{3}$ are bounded such as:

(17) $$\begin{array}{cc} |u_{1}| & =max(M_{13},M_{23},0.707\left(M_{13}+M_{23}\right))\\ |u_{2}| & =max(M_{13},M_{23},0.707\left(M_{13}+M_{23}\right))\\ |u_{3}| & =M_{33} \end{array}$$

**Figure 9:**
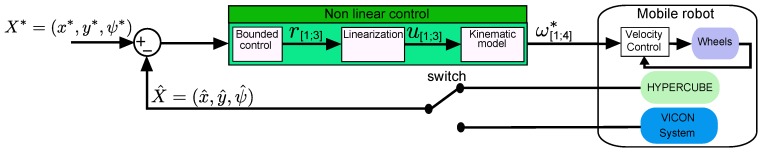
Block diagram of the robot autopilot. The position and heading are controlled in closed-loop. The mobile robot is equipped with HyperCube

Therefore, the angular speed of each wheel $\omega_{i}$ of the mobile robot is bounded such as the following:

(18) $$\begin{array}{cc} |\omega_{1}| & =\frac{1}{R_{w}}\left(\left(L+l\right)M_{33}\right)\\ |\omega_{2}| & =\frac{1}{R_{w}}\left(2\cdot max\left(M_{13},\;M_{23},\;0.707\left(M_{13}+M_{23}\right)\right)+\left(L+l\right)M_{33}\right)\\ |\omega_{3}| & =\frac{1}{R_{w}}\left(2\cdot max\left(M_{13},\;M_{23},\;0.707\left(M_{13}+M_{23}\right)\right)+\left(L+l\right)M_{33}\right)\\ |\omega_{4}| & =\frac{1}{R_{w}}\left(\left(L+l\right)M_{33}\right) \end{array}$$

where $M_{ij},\;\forall i,j\;\in \;\left\{1,2,3\right\}$ is the saturation parameter as defined in (13a). Figure 9 presents the block diagram of the system. The position and heading of the mobile robot are controlled in closed loop. It is worth noting that:

- The nonlinear control block computes each angular reference speed $\omega_{i}^{*}$ for the wheel *i*. The control law minimizes the error between the reference position $X^{*}$ and the position estimate $\hat{X}$.
- The angular speed of each wheel is controlled in closed loop using a local proportional integral controller (PI).
- The estimated position and heading of the mobile robot collected in the vector $\hat{X}=\left(\hat{x},\hat{y},\hat{\phi }\right)$ are provided by HyperCube or the Vicon motion capture system .

#### 5.2.3. Implementation of the Indoor Localization for the Mobile Robot

**Figure 10:**
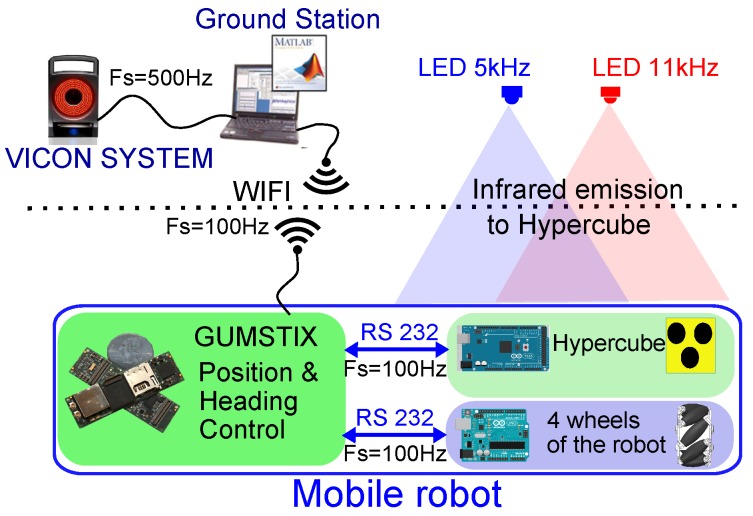
Sketch diagram of the implementation of the indoor localization system for mobile robots. The Arduino Mega 2560 gets the analog signals provided by the demodulation board (Arduino shield, see Figure 2) and the output voltage of each photodiode $Ph_{i}$ of HyperCube. The Arduino 380 board is devoted to control the angular velocity of each wheel.

To evaluate the performances of the indoor localization, the setup detailed in Figure 10 is composed of the following:

- The Vicon motion capture system featuring sub-millimetric accuracy. It provides the localization estimation of the mobile robot. The motion capture data are used for comparison purposes.
- The ground station connected to the Vicon system runs Matlab/Simulink^®^ and QUARC^®^ software programs. The nonlinear control law of the mobile robot presented in Section 5.2.2 is designed with Matlab/Simulink^®^ and compiled. The program is transferred via WIFI radio link to the Gumstix microcontroller embedded on the mobile robot. The control algorithm runs onboard the robot.

The mobile robot aims at tracking a desired trajectory using HyperCube in a feedback control loop. An Arduino 380 board controls in closed-loop the angular velocity of each wheel. An Arduino Mega 2560 board is connected to the custom-made demodulation board of HyperCube which provides analog signals. The analog processing of the photodiode’s output signal is depicted in Figure 4. The Gumstix board uses the estimation of the robot position $\hat{X}=\left(\hat{x},\hat{y},\hat{\phi }\right)$ for control purpose.

**Figure 11:**
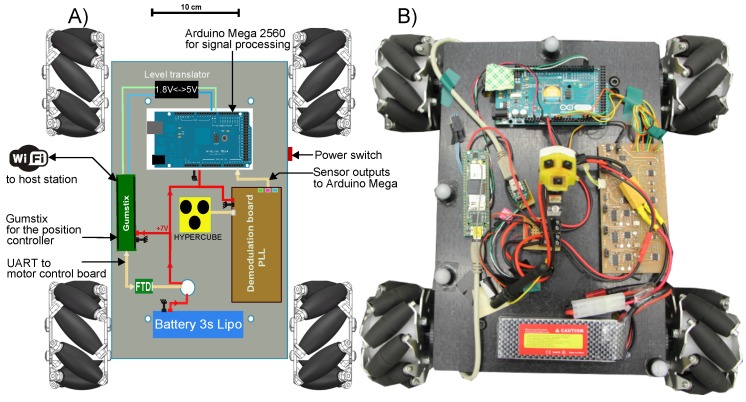
Top view of the mobile robot: (**A**) schematic of the embedded hardware; and (**B**) picture of the mobile robot used for the experiments.

Figure 11A gives a schematic of the hardware embedded on the mobile robot for indoor localization using HyperCube. Figure 11B shows the hardware implementation and HyperCube mounted onboard the mecanum wheeled omni-directional robot. Four reflective markers make the mobile robot visible by the Vicon system.

#### 5.2.4. Application to the 2D Localization

The aim of this section is twofold: (i) using HyperCube in open loop, reconstruct the trajectory (x,y) of the mobile robot in the coverage area presented in Figure 7; and (ii) using HyperCube in closed loop, track a desired trajectory. For that purpose, the following experiments are performed.

#### 5.2.5. Validation of the Nonlinear Control Law for Trajectory Tracking

**Figure 12:**
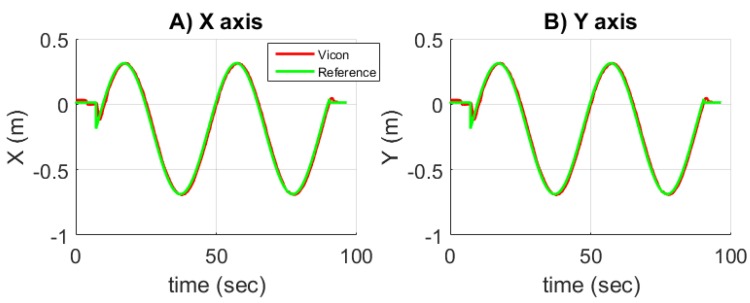

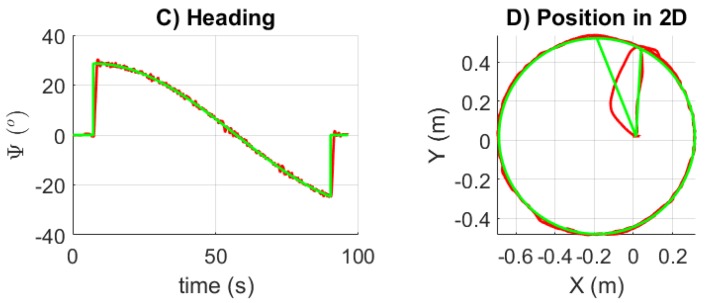
(**A**–**C**) plots of the position and orientation of the mobile robot. The accurate measurements provided here only by means of the Vicon system provide to the sliding mode nonlinear controller the measured robot’s positions (*x*,*y*) and heading. The path is compared to the desired trajectory.

The Vicon cameras provide to the nonlinear controller detailed in Section 5.2.2 the accurate localization and orientation of the robot in real-time. The mobile robot moves on a circular path of diameter 1 m. In the same time, the desired orientation ψ is a sinus. The results are given in Figure 12 where Figure 12A–C shows in red the positions (x,y) and the heading ψ versus time of the mobile robot compared to the reference in green. Figure 12D plots *y* versus *x*. Using accurate estimations of position and heading, the mobile robot follows the desired trajectory with precision without overshoot. This result validates the good performances of the sliding mode nonlinear control law.

#### 5.2.6. Trajectory Reconstruction Using HyperCube

**Figure 13:**
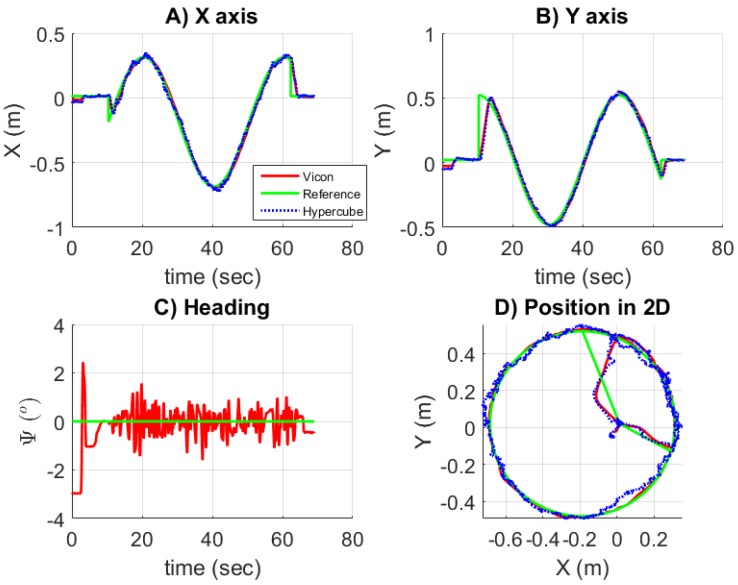
Plots of the position and orientation of the mobile robot. The accurate measurements provided by the Vicon system feed the sliding mode nonlinear controller. The trajectory is reconstructed using HyperCube and the IR LED that flickers at 11 kHz. The closed-loop control of the robot’s positions and heading is only based here on the measurements provided by the Vicon system.

While keeping the circular path of diameter 1 m as reference, the desired orientation ψ is regulated at zero. The mecanum wheeled omni-directional robot maintains its orientation along the path. In this experiment, the trajectory is reconstructed using the measurements provided by HyperCube and only one IR LED that flickers at 11 kHz. However, the estimated positions provided by HyperCube were not used to control in closed-loop the robot. Figure 13 shows the results of the reconstruction. In Figure 13A,B, the position estimations in *X* and *Y* are plotted versus time and compared to the ground truth provided by the Vicon system. Figure 13C presents the heading ψ which is maintained at zero. There is no curve for HyperCube since HyperCube is not used to estimate the heading. Figure 13D depicts the *XY* graph. Satisfactory trajectory reconstruction can be verified by comparing the actual path of the mobile robot to the estimated one given by HyperCube.

**Figure 14:**
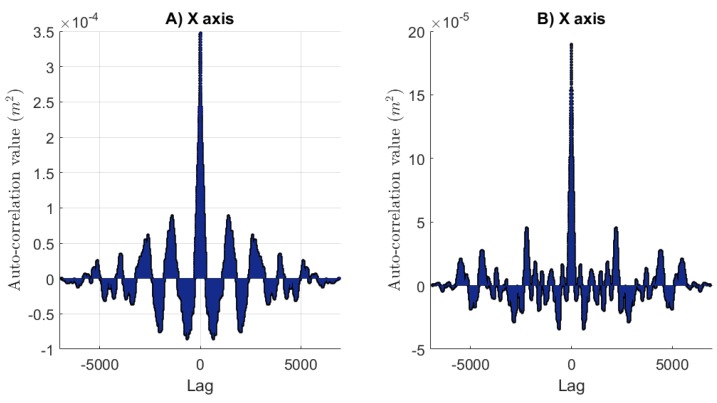
Plots of the autocorrelation functions of the residuals for each axis *X* and *Y*.

One can see in Figure 14A,B the plots of the autocorrelation functions of the residuals. The noise for each axis has nearly white characteristics.

**Figure 15:**
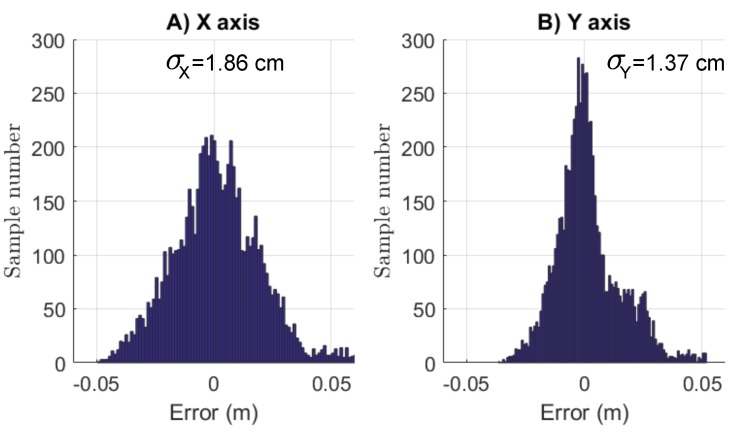
Histograms of the localization error in 2D using HyperCube. The standard deviations for the *X* and *Y* axis are $\sigma_{X}=1.86$ cm and $\sigma_{Y}=1.37$ cm for H=2 m. Only one IR LED that flickers at 11 kHz is used.

Moreover, given H=2 m, the precision obtained reaches a standard deviation as small as 1.86 cm for *X* and 1.37 cm for Y (see Figure 15). Looking accross the precision of the reconstruction, the mobile robot was then controlled by means of HyperCube.

#### 5.2.7. Robot Closed-Loop Control Based on HyperCube

**Figure 16:**
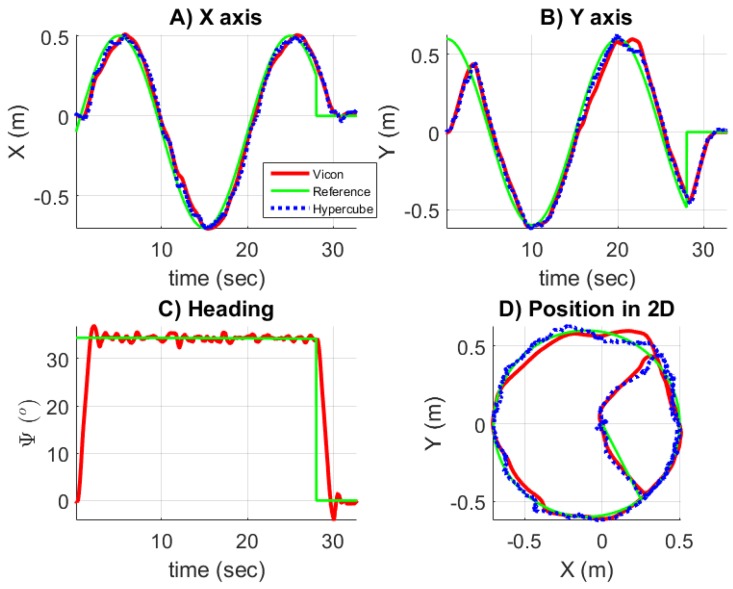
Plots of the position and orientation of the mobile robot while the measurements provided by HyperCube are used in closed loop and the heading is given by the Vicon cameras. Only one IR LED that flickers at 11 kHz is used and H=2 m.

In this section, the goal was to use the measurements provided by HyperCube to control in closed-loop the linear positions (*x*, *y*) of the robot. The current version of the sensor can only estimate the positions but not the rotations. For a robotic application using aerial robots for instance, HyperCube could be associated to a stabilizing gimbal system to reduce the effects of the variations of roll and picth angles which could affect the measurements. In the present application, the orientation ψ is given by the Vicon cameras. If we denote $x_{sensor}$ and $y_{sensor}$ the coordinates of the mobile robot in the local frame, the estimates of position $\hat{x}$ and $\hat{y}$ in the global frame are obtained by applying the rotation matrix as following:

(19) $$\left[\begin{array}{c} \hat{x}\\ \hat{y}\\ \hat{\psi } \end{array}\right]=\left[\begin{array}{ccc} \cos(\hat{\psi }) & -\sin(\hat{\psi }) & 0\\ \sin(\hat{\psi }) & \cos(\hat{\psi }) & 0\\ 0 & 0 & 1 \end{array}\right]\left[\begin{array}{c} x_{sensor}\\ y_{sensor}\\ \psi_{vicon} \end{array}\right]$$

where $\psi_{vicon}$ is the estimation of the orientation given by the Vicon cameras. Figure 16 shows the results while the sensor is used in closed loop. The orientation ψ is estimated with the motion capture system. Figure 16A,B superimposes the positions *x* and *y* measured by HyperCube (blue curve) and by the Vicon system (red curve). Figure 16C presents the regulation of the heading versus time. Since the orientation is given by the Vicon cameras and not by HyperCube, there is no curve related to HyperCube in Figure 16C. Once equipped with HyperCube, the experimental results show that HyperCube can be suitable to make the mobile robot follow faithfully the reference trajectory. Therefore, the performance of the HyperCube sensor have been validated indoors by using only one flickering IR LED. A limitation of this solution is the need of the orientation ψ provided by the Vicon system. However, for this kind of application, onboard sensors such as a low-cost inertial measurement unit (IMU) for instance in [20], magnetometer as proposed in [21] or odometry could be used to get the heading in real time.

## 6. Conclusions

In this paper, we proposed a novel positioning system for indoor application which can measure the angular position of a moving optical sensor, HyperCube. The latter was coupled to an Arduino-compatible demodulator through a custom-made shield board. Based on infrared light emission, the proposed solution for indoor localization spotlights the following features: (i) a minimalistic sensor in terms of small size (10 cm${}^{3}$), light weight (6 g) and low power consumption (0.4 W for the sensor and the analog demodulation board); (ii) a fast analog signal processing and a digital processing implemented on an Arduino microcontroller (difference over the sum); and (iii) an online accurate position estimation in 2D.

We have shown that the proposed sensor was able to estimate the position in 2D at a distance of 1.5 m from the LEDs with an accuracy as small as 1-cm at a sampling frequency of 100 Hz using only one IR LED flickering at 17 kHz. We also proposed a robotic application which consisted in localizing a moving mecanum wheeled omnidirectional robot indoors. For a trajectory reconstruction purpose, the precision of the position estimation reached good performances with a standard deviation as small as 1.86 cm for *X* and 1.37 cm for *Y* with only one flickering IR LED placed at 2 m above the robot. To show the performance of HyperCube, the sensor was implemented in the position feedback control loop to make the mobile robot able to track a reference trajectory (a circle of 1 m in diameter). It turned out that the mobile robot followed the desired path faithfully, validating that the indoor local positioning system using flickering IR LEDs is a reliable and an efficient solution.

To further improve the performance of the proposed system, the sensor measurements provided by HyperCube will be fused with other sensors such as IMU to improve the position estimation. The height could also be estimated using two IR LEDs at two different frequencies. Moreover, one could investigate the estimation of the heading ψ using several IR LEDs at the same time.

## Supplementary Materials

The following are available online at http://www.mdpi.com/1424-8220/17/11/2518/s1, Video S1: Robot closed-loop control based on HyperCube.

## Abbreviations

The following abbreviations are used in this manuscript:

AGV	Automated Guided Vehicle
CCD	Charge Couple Device
CMOS	Complementary Metal Oxide Semiconductor
GPS	Global Positioning System
IMU	Inertial Measurement Unit
IR	Infrared
LED	Light Emitting Diode
LIDAR	LIght Detection And Ranging
PD	Photodiode
WIFI	Wireless Fidelity
